# Multiple sexual partners and condom use among 10 - 19 year-olds in four districts in Tanzania: What do we learn?

**DOI:** 10.1186/1471-2458-11-490

**Published:** 2011-06-22

**Authors:** Amon Exavery, Angelina M Lutambi, Godfrey M Mubyazi, Khadija Kweka, Godfrey Mbaruku, Honorati Masanja

**Affiliations:** 1 Ifakara Health Institute (IHI), Plot 463, Kiko Avenue, off Old Bagamoyo Road, Mikocheni P.O Box 78373, Dar es Salaam, Tanzania; 2 National Institute for Medical Research (NIMR), Centre for Enhancement of Effective Malaria Interventions (CEEMI) in Collaboration with the National Malaria Control Programme (NMCP) under the Ministry of Health and Social Welfare, P.O Box 9653, Dar es Salaam, Tanzania

## Abstract

**Background:**

Although some studies in Tanzania have addressed the question of sexuality and STIs among adolescents, mostly those aged 15 - 19 years, evidence on how multiple sexual partners influence condom use among 10 - 19 year-olds is limited. This study attempts to bridge this gap by testing a hypothesis that sexual relationships with multiple partners in the age group 10 - 19 years spurs condom use during sex in four districts in Tanzania.

**Methods:**

Secondary analysis was performed using data from the *Adolescents Module *of the cross-sectional household survey on Maternal, Newborn and Child Health (MNCH) that was done in Kigoma, Kilombero, Rufiji and Ulanga districts, Tanzania in 2008. A total of 612 adolescents resulting from a random sample of 1200 households participated in this study. Pearson Chi-Square was used as a test of association between multiple sexual partners and condom use. Multivariate logistic regression model was fitted to the data to assess the effect of multiple sexual partners on condom use, having adjusted for potential confounding variables. STATA (10) statistical software was used to carry out this process at 5% two-sided significance level.

**Results:**

Of the 612 adolescents interviewed, 23.4% reported being sexually active and 42.0% of these reported having had multiple (> 1) sexual partners in the last 12 months. The overall prevalence of condom use among them was 39.2%. The proportion using a condom at the last sexual intercourse was higher among those who knew that they can get a condom if they want than those who did not. No evidence of association was found between multiple sexual partners and condom use (OR = 0.77, 95% CI = 0.35 - 1.67, P = 0.504). With younger adolescents (10 - 14 years) being a reference, condom use was associated with age group (15 - 19: OR = 3.69, 95% CI = 1.21 - 11.25, P = 0.022) and district of residence (Kigoma: OR = 7.45, 95% CI = 1.79 - 31.06, P = 0.006; Kilombero: OR = 8.89, 95% CI = 2.91 - 27.21, P < 0.001; Ulanga: OR = 5.88, 95% CI = 2.00 - 17.31, P = 0.001), Rufiji being a reference category.

**Conclusion:**

No evidence of association was found between multiple sexual partners and condom use among adolescents in the study area. The large proportion of adolescents who engage in sexual activity without using condoms, even those with multiple partners, perpetuates the risk of transmission of HIV infections in the community. Strategies such as sex education and easing access to and making a friendly environment for condom availability are important to address the risky sexual behaviour among adolescents.

## Background

The incidence of Acquired Immune Deficiency Syndrome (AIDS) cases due to Human Immunodeficiency Virus (HIV) continues to rise worldwide [1,2], with nearly 50 percent of all new infections on the globe occurring among 15 - 24 year-olds [2]. Sub-Saharan Africa is the region hardest hit by the HIV/AIDS pandemic than any other in the world. In 2007, about 22.5 million people were living with HIV/AIDS, with approximately 1.7 million people newly infected with the virus [3]. The 2007-08 Tanzania HIV/AIDS and Malaria Indicator Survey (THMIS) reported an estimated HIV/AIDS prevalence of 6% among Tanzanian adults aged 15 - 49 [4]. This figure is lower than the 2003 country's HIV/AIDS prevalence of 8.8% which was higher than the overall prevalence of 7.5%) in Sub-Saharan Africa in 2003 [2].

Person-to-person sexual contact is known to be a primary pathway through which sexually transmitted infections (STIs) including HIV/AIDS spread [5]. Cultural and environmental contexts play an important role in variations in sexual practices across regions and societies [6]. In Tanzania, like elsewhere in Sub-Sahara Africa, adolescents (defined by WHO as persons in the age group 10 - 19 years [7]) are greatly vulnerable to STIs as they engage in risky sexual behaviors, such as unprotected sex, multiple sexual partners and young age at sexual debut [8,9]. Recent statistics from Tanzania show that 32% of the adolescents aged 10 - 19 years were sexually active, with 15% of them reporting multiple sexual partners. Vaginal sex was the prominent form of sexual practices, outweighing anal, masturbation and oral sex [8]. Infidelity which immediately translates into multiple sexual partners has been documented as a key medium through which many STIs including HIV/AIDS spread.

Nevertheless, proper and consistent condom use has been acknowledged to be effective towards successful prevention of STIs [5,10,11], including 80 - 90% of heterosexual transmission of HIV [12]. Despite this reality, prevalence of condom use in Sub-Saharan Africa is still low and inconsistent [12] - less than 15% in Tanzania, Malawi and Ethiopia among those aged 15 - 19 years [13]. Objections to condoms due to allegations that condoms inhibit the enjoyment of sex, condoms cause sores on a penis, condoms come off inside a woman and it is a sin to waste semen and prevent pregnancy are widespread in Sub-Saharan Africa [14,15]. From a religious point of view, there are also sanctions against condom use among Catholics and some Evangelists asserting that it fuels promiscuity [16]. Similarly, promoting condoms is considered as encouraging sexual acts among adolescents [10].

On the other hand, sexual partners tend to use condoms at first, only to stop later in subsequent sexual contacts (without testing for HIV) when their relationship deepens [14] due to claims that using a condom connotes distrust or absence of intimate love [17,18].

A study conducted in Mwanza, Tanzania among primary and secondary school students aged 12 - 19 years, revealed a condom prevalence of 30% and a lower rate of STIs among those who were well informed about STIs, HIV/AIDS and used condoms [19]. Another study in the same country reported reasons youths give for not using condoms as trusting a partner, not having condoms, ineffectiveness of condoms in preventing HIV transmission and reduced sexual pleasure [20]. Among secondary school and college students aged 16 - 24 years in Tanzania, it was observed that 35% of those who had multiple sexual partners in the previous year did not always use condoms [21], whereas in Angola, youths aged 15 - 24 years with multiple sexual partners were consistent condom users [22].

Tanzania's Health Policy stipulates that the Ministry of Health and Social Welfare (MoHSW) will promote youth-friendly services to improve access to reproductive health information and services [23], but scale-up of youth-friendly interventions has been proven challenging because of structural constraints and broader social norms [24] and coverage of such services is therefore still low [13]. This highlights the importance of finding alternatives to address the situation.

Although some studies in Tanzania have addressed the question of sexuality and STIs among adolescents, mostly those aged 15 - 19 years, there is limited evidence on how multiple sexual partners influence condom use amongst adolescents. In this study, we thus test a hypothesis that being in sexual relationships with multiple sexual partners among 10 - 19 year-olds spurs condom use during sex.

## Methods

### Study design and study area

The data for this study was collected as part of a larger household survey on Maternal, Newborn and Child Health (MNCH). This was a cross-sectional study that was carried out by the Ifakara Health Institute (IHI) in partnership with the WHO (Regional Office for Africa) and the MoHSW in four districts in Tanzania mainland where the IHI's Empower project is being implemented, namely Kigoma, Kilombero, Rufiji and Ulanga. "Multiple sexual partners" was defined as having had more than one sexual partner in the last 12 months preceding the interview. Condom use was measured dichotomously as having used/not used a condom at the last sexual intercourse for either partner. Following the standard age categorization, two groups of the study participants' age were formed as 10 - 14 and 15 - 19 years and referred to as *younger adolescents *and *older adolescents *respectively [25,26].

### Sampling and study population

Sampling was done systematically using the CSurvey software. The sample size was determined depending on the indicators to be measured on population demographics (e.g. household size, fertility) and on local prevalence of selected conditions such as diarrhoea, cough with rapid breathing and fever. A total of 1200 random households, being 300 households selected randomly from each district were sampled.

The target population was school-aged adolescents (10 - 19 years) and this survey was conducted during their mid-year holiday. In addition, sampled households were informed in advance by their respective community leaders to be available at home for an interview on agreed day. Respondents were childbearing-aged women with a child aged < 5 years and adolescents. Information regarding children aged < 5 years was given voluntarily by a child's mother or guardian after signing a written informed consent. Adolescents aged 18 and above signed the consent by themselves after which they were interviewed. For the minors (under 18), the consent was sought from their parents/guardians. The response rate of this household survey on MNCH was as high as 94%.

### Data collection and Management

Data collection took place between June and July 2008 through face-to-face interviews, using a questionnaire that was organized in different non-overlapping modules. Personal Digital Assistants (PDAs) were used for data collection and then the data were being synchronized into a laptop computer and backed up daily. The current study analyzed the *Adolescents Module *of the questionnaire, whereby 612 adolescents aged 10 - 19 years were interviewed.

### Data analysis

We analyzed some demographic variables particularly sex, age and place of residence. The analysis focused on five key questions: 1. Have you ever had sex? 2. The last time you had sex, did you or your partner use condom? 3. Have you ever had more than one sexual partner in the last 12 months? 4. Have you ever had a HIV test? 5. If you wanted, could you get a condom?

The data were first analyzed descriptively to obtain summary statistics of the study participants. This was followed by testing for associations between condom use and the explanatory variables using Chi-square and student t-tests for categorical and continuous variables respectively. Multivariate logistic regression model was finally fitted to the data to assess the effect of multiple sexual partners on condom use, controlling for other potential confounding variables. This process was carried out using STATA (version 10) statistical software at 5% two-sided significance level.

## Results

**Figure 1:**
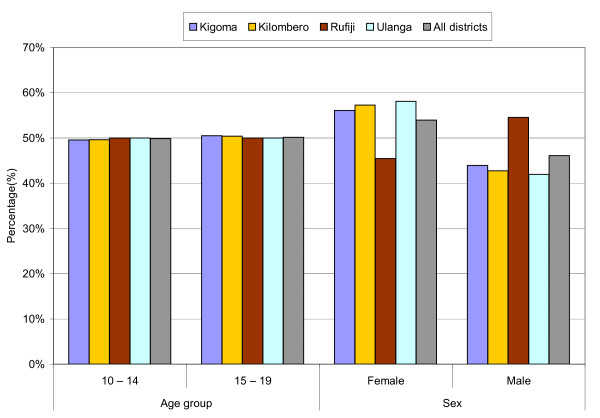
**Percentage distribution of adolescent's age group and sex by district of residence in Tanzania, 2008 (N = 612)**.

Six hundred and twelve Tanzanian adolescents participated in this study. Of these, 330 (53.9%) were female and the rest were male adolescents. Their mean age was 14.3 years with a standard deviation of 2.50. About half (49.8%) of them were in the 10 - 14 and 15 - 19 years age group each. In terms of district of residence, the sample was made up of Kigoma (17.5%), Kilombero (21.4%), Rufiji (28.8%) and Ulanga (32.3%) (Table 1). Age and sex distribution by district of residence is presented in Figure 1. The proportion of adolescents in each age group (i.e. 10 - 14 and 15 - 19) was around 50% across the four districts. With regards to sex, the proportion of female adolescents was higher (at least 56%) than that of males in Kigoma, Kilombero and Ulanga, but conversely, the proportion of male adolescents (55%) was higher than that of females in Rufiji.

**Table 1:** Demographic characteristics of the study sample, 2008. (N = 612)

Variable	n (%)
**Sex**	
Female	330 (53.9%)
Male	282 (46.1%)
**Age (years)**	
10 - 14	305 (49.8%)
15 - 19	307 (50.2%)
**District of residence**	
Kigoma	107 (17.5%)
Kilombero	131 (21.4%)
Rufiji	176 (28.8%)
Ulanga	198 (32.3%)

n = Number of respondents for variable category

**Figure 2:**
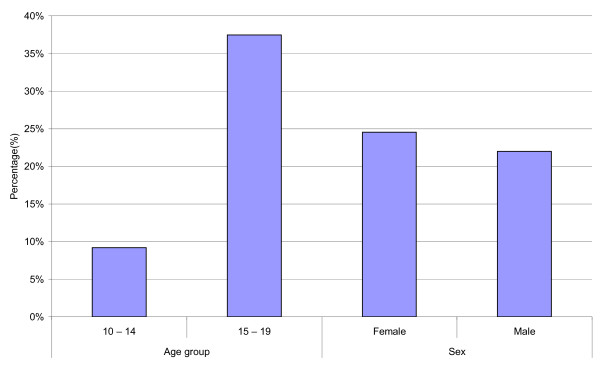
**Percentage distribution of 143 sexually active adolescents by age group and sex in four districts in Tanzania, 2008**.

One hundred forty three (23.4%) adolescent respondents (24.6% males and 22.0% females) reported being sexually active. Of these, 42.0% reported having had multiple sexual partners in the last 12 months. Among those aged 15 - 19 years, 37.5% were sexually active and the corresponding figure among the 10 - 14 year-olds was 9.4% (Figure 2). Data regarding the sexually active respondents were further analyzed to investigate associations between multiple sexual partners and condom use.

### Prevalence of condom use

**Figure 3:**
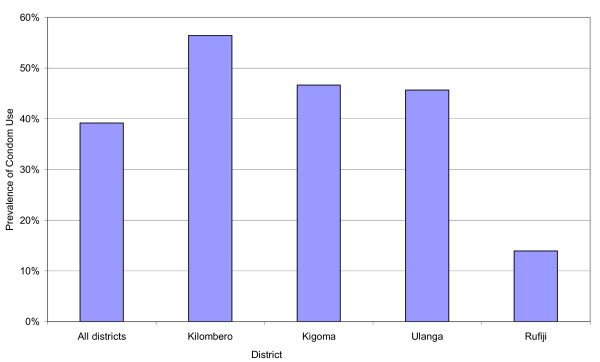
**Prevalence of condom use at the last sexual intercourse among 143 sexually active adolescents by district of residence in Tanzania, 2008**.

The prevalence of condom use at the last sexual intercourse among the sexually active respondents in the study area ranged from 14% to 56%, with an overall prevalence of 39%. The prevalence of condom use was lowest in Rufiji and highest in Kilombero. Kigoma (47%) and Ulanga (46%) had similar prevalence of condom use (Figure 3).

Table 2 presents a test of associations between condom use and a set of explanatory variables. Condom use was found to be independent of multiple sexual partners (P = 0.863), hence failing to reject the null hypothesis of no association between condom use and multiple sexual partners. Condom use was however associated with age group (P = 0.010), district of residence (P = 0.001), knowing that it is possible to obtain a condom if one wants (P = 0.049) and knowing that it is possible to get a HIV test if one wants (P = 0.041) (Table 2).

**Table 2:** Distribution of factors associated with condom use at the last sexual intercourse among 143 sexually active adolescents in four districts in Tanzania, 2008.

Variable	Condom use at last sex		P-value*
		
	YES	NO	
	n (%)	n (%)	
**Multiple sexual partners in the last 12 months**			0.863
No	33 (39.8%)	50 (60.2%)	
Yes	23 (38.3%)	37 (61.7%)	
**Sex**			0.658
Female	33 (40.7%)	48 (59.3%)	
Male	23 (37.1%)	39 (62.9%)	
**Age (years)**			**0.010**
10 - 14	5 (17.9%)	23 (82.1%)	
15 - 19	51 (44.4%)	64 (55.6%)	
**District of residence**			**0.001**
Kigoma	7 (46.7%)	8 (53.3%)	
Kilombero	22 (56.4%)	17 (43.6%)	
Rufiji	6 (14.0%)	37 (86.0%)	
Ulanga	21 (45.7%)	25 (54.4%)	
**If you wish, could you get a condom?**			0.049**
No	2 (14.3%)	12 (85.7%)	
Yes	54 (41.9%)	75 (58.1%)	
**Do you know that it is possible to get a HIV test if you wish?**			0.041**
No	3 (16.7%)	15 (83.3%)	
Yes	53 (42.4%)	72 (57.6%)	

* Based on Pearson Chi-Square test

** Fisher's exact test for a contingency table with an expected cell count of < 5

Results of the multivariate logistic regression analysis are presented in table 3. Having adjusted for potential confounding variables, condom use remained independent of multiple sexual partners. However, adolescents reporting multiple sexual partners were 23% less likely to use a condom at the last sexual intercourse compared to those without multiple partners, but this effect was not statistically significant (OR = 0.77, 95% CI = 0.35 - 1.67, P = 0.504).

**Table 3:** Multivariate logistic regression analysis of the association between multiple sexual partners and condom use at the last sexual intercourse among 143 sexually active adolescents in four districts in Tanzania, 2008.

Variable	n	Condom use at last sex		
		
		OR	(95% CI)	P-value
**Multiple sexual partners in the last 12 months**				
No	83	Ref.		
Yes	60	0.77	(0.35 - 1.67)	0.504
**Age (years)**				
10 - 14	28	Ref.		
15 - 19	115	3.69	(1.21 - 11.25)	**0.022**
**District of residence**				
Rufiji	43	Ref.		
Kigoma	15	7.45	(1.79 - 31.06)	**0.006**
Kilombero	39	8.89	(2.91 - 27.21)	**< 0.001**
Ulanga	46	5.88	(2.00 - 17.31)	**0.001**
**If you wish, could you get a condom?**				
No	14	Ref.		
Yes	129	4.00	(0.79 - 20.29)	0.095

n = Number of respondents for variable category; OR = Odds ratio; CI = Confidence interval; Ref. = Reference category.

The influence of multiple sexual partners on condom use is adjusted for age, district of residence and whether or not one can obtain a condom if he/she wants. Factors that do not appear in this table (regardless of their significance level in the test of associations in table 2) presented immaterial effect on condom use and this remained impervious when the log likelihood ratio test was used. Since this was somewhat a small sample, the log likelihood ratio test was used to select variables for the multivariate model (rather than relying on the P < 0.2 criterion), except multiple sexual partnerships which was the exposure variable of interest.

Independent predictors of condom use at the last sexual intercourse were age group and district of residence. Condom use at the last sexual intercourse was nearly 4 times higher among 15 - 19 year-olds compared to the 10 - 14 year-olds (OR = 3.69, 95% CI = 1.21 - 11.25, P = 0.022). In terms of district of residence, Rufiji was made a reference category and the rest of the districts were compared to it. The odds of condom use in Kigoma was approximately 7 times as high as that observed in Rufiji (OR = 7.45, 95% CI = 1.79 - 31.06, P = 0.006). Similarly in Kilombero, the odds of condom use was approximately 9 times as high as that observed in Rufiji (OR = 8.89, 95% CI = 2.91 - 27.21, P < 0.001). It was also about 6 more likely among adolescents in Ulanga than those in Rufiji (OR = 5.88, 95% CI = 2.00 - 17.31, P = 0.001). The likelihood of condom use among adolescents in Kigoma, Kilombero and Ulanga was statistically similar.

In addition, we re-fitted the multivariate logistic regression model restricted to the older adolescents (Table 4) and found similar results to those presented in table 3.

**Table 4:** Additional multivariate logistic regression analysis restricted to 115 sexually active older adolescents (15 - 19 years) of the association between multiple sexual partners and condom use at the last sexual intercourse in four districts in Tanzania, 2008.

Variable	n	Condom use at last sex		
		
		OR	(95% CI)	P-value
**Multiple sexual partners in the last 12 months**				
No	64	Ref.		
Yes	51	0.68	(0.30 - 1.57)	0.369
**District of residence**				
Rufiji	34	Ref.		
Kigoma	11	9.21	(1.97 - 42.98)	**0.005**
Kilombero	33	8.35	(2.58 - 27.09)	**< 0.001**
Ulanga	37	5.03	(1.63 - 15.46)	**0.005**
**If you wish, could you get a condom?**				
No	8	Ref		
Yes	107	3.02	(0.54 - 16.81)	0.206

n = Number of respondents for variable category; OR = Odds ratio; CI = Confidence interval; Ref. = Reference category.

Diagnostic tests were carried out to assess the adequacy of the logistic regression models presented in table 3 and table 4. The Hosmer-Lemeshow goodness of fit test at the generally recommended number of groups [27] showed no evidence of lack of fit in the models (P > 0.05). Moreover, no statistical interaction (effect modification) was observed.

## Discussion

This study has shown that condom use among adolescents in the four districts in Tanzania, namely Kigoma, Kilombero, Rufiji and Ulanga is not associated with multiple sexual partners. We found evidence of association of condom use by age group and by district of residence.

Despite the fact that proper and consistent condom use prevents STIs successfully [10,11], a significant proportion of the adolescents in the study area remains at risk of contracting STIs and unintended pregnancies. This is evident from the present study which indicates that among the sexually active adolescents, 61% did not use condoms at the last sexual intercourse and about 42% of them reported having had sex with multiple partners in the last 12 months. Meanwhile, more than three-fifth of those reporting multiple partners did not use condoms at their last sexual intercourse.

The likelihood of condom use was higher among older adolescents (15 - 19 year-olds) compared to the younger ones (10 - 14 year-olds), possibly due to knowledge differentials about transmission and prevention of STIs as the former may have had longer exposure to sensitization messages. This observation is consistent with others from other studies [28,29]. However, the prevalence of multiple sexual partners was similar among the younger and the older adolescents. This is probably linked to the fact that both are on a transition into adulthood, thus experiencing much of physical, emotional and psychological changes and also being influenced by peer behaviours [30].

The lower level of condom use found in Rufiji compared to other districts may be partly attributable to the fact that rural residences (Rufiji being the most) are often correlated with lower levels of condom use and also inequality in condom accessibility [31]. It may also be linked to variations in cultural norms and differentials in access to information, communication and education [32].

Although parents or guardians can be the best entry point to reach adolescents, the fact that sex-related matters are very sensitive pose a great challenge to discussing them at home. In many communities, premarital sex is culturally or religiously forbidden [16] and youths engaged in it are considered to be misbehaving. The truth however is that a good number of adolescents engage in sexual activities stealthily and in fear of condemnation and mistrust. Therefore, not only the unfriendly environment for accessing condoms and other reproductive health services that matters, but also adolescents are afraid to buy condoms, especially in open outlets [33]. This calls for adolescent-friendly sex and reproductive health interventions which fully respond to such contextual limitations.

A separate analysis (results not presented) carried out to identify factors associated with multiple sexual partners revealed that male adolescents were two times more likely than females to report multiple sexual partners. This observation is in agreement with findings from other studies [34-36]. We also observed (Table 2) that 41% of all sexually active female adolescents, used condoms at their last sexual intercourse. The corresponding figure for male adolescents was 37%, but the difference was not statistically significant (P = 0.658). This slightly higher rate of condom use observed among female adolescents can partly be explained by fear of getting pregnant. From this, it appears somewhat that male adolescents are more likely than their female counterparts to engage in risky sexual behaviours. Thus, context-specific interventions are important in order to reduce the chances of transmission of STIs and unintended pregnancies among adolescents.

### Limitations

Some important variables including religion, condom brand types, education level, frequency of sexual intercourse, whether or not sex was coercive and some household variables such as socio-economic status, that could further explain the relationship between multiple sexual partners and condom use were not available for this study. It should also be noted that due to the snapshot nature of cross-sectional studies, we cannot draw causal inferences from the findings of this study. Similarly, we cannot assert these findings as globally generalizable, because being in sexual relationship with multiple partners among adolescents has been found in other areas to be associated with condom use [22]. Further research however is needed to assess if the findings apply to other regions of Tanzania.

The study relied on self-reported information on sexual behaviours which is often unreliable [37,38], difficult to verify and subject to recall bias or deliberate omission of some sensitive information.

However, all possible precautionary measures were taken to reduce biases in this study. Before the data collection began, a rigorous interviewer training was held for five days, in which the survey instrument was reviewed. The training also covered other important aspects such as basic interview techniques (e.g. probing, establishing rapport) and simulated interviews. The training was conducted in *Swahili *- Tanzania's national language - and the survey instrument was also translated from English into this language which is well understood and widely spoken locally. The instrument was then pretested in nearby villages with respondents who were similar to those who participated in the main survey study. All interviews during the pretest and the main survey were conducted in *Swahili*. A few changes were made to the survey instrument following the pretest feedback and ultimately, data collection commenced immediately.

## Conclusions

We found no evidence of association between multiple sexual partners and condom use among adolescents in the four districts in Tanzania. A notable number of adolescents are engaged in sexual activity and a few use condoms during sex even those with multiple partners, probably due to limited knowledge on safe sex, cultural norms, unfriendly environment for condom accessibility etc thus exposing them to the risk of contracting STIs including HIV/AIDS and unintended pregnancies.

### Recommendations

Sex and reproductive health education remains one of the very important strategies to address risky sexual behaviour among adolescents. Expanding coverage of adolescent reproductive health services need to be prioritized. For both the younger and the older adolescents, it is very crucial to emphasize condom use during every sexual encounter and consider limiting the number of sexual partners to one uninfected and faithful partner. Abstinence remains the most reliable method of prevention against the STIs.

## Competing interests

The authors declare that they have no competing interests.

## Authors' contributions

AE envisioned the problem, performed data analysis and wrote the manuscript drafts. AML contributed to data analysis and reviewed the manuscript drafts. GM, HK, HM and GMM contributed to study designing and read through the manuscript drafts for fine-tuning. All the authors read and approved the final draft of this manuscript.

## Pre-publication history

The pre-publication history for this paper can be accessed here:

http://www.biomedcentral.com/1471-2458/11/490/prepub
